# Lactic Acidosis Related to Pharmacotherapy and Human Diseases

**DOI:** 10.3390/ph15121496

**Published:** 2022-11-30

**Authors:** Christian Zanza, Valentina Facelli, Tastiana Romenskaya, Maria Bottinelli, Giorgia Caputo, Andrea Piccioni, Francesco Franceschi, Angela Saviano, Veronica Ojetti, Gabriele Savioli, Yaroslava Longhitano

**Affiliations:** 1 Foundation “Ospedale Alba-Bra Onlus,” Department of Emergency Medicine, Anesthesia and Critical Care Medicine, Michele and Pietro Ferrero Hospital,12060 Verduno, Italy; 2 Department of Emergency Medicine, Policlinico Agostino Gemelli, Catholic University of Sacred Heart, 00168 Rome, Italy; 3 Department of Anesthesia and Critical Care Medicine, Azienda Ospedaliera “SS Antonio e Biagio e C. Arrigo”, 15121 Alessandria, Italy; 4 Department of Physiology and Pharmacology, Sapienza University of Rome, 00185 Rome, Italy; 5 Emergency Department, IRCCS Fondazione Policlinico San Matteo, 27100 Pavia, Italy; 6 PhD School in Experimental Medicine, Department of Clinical-Surgical, Diagnostic and Pediatric Sciences, University of Pavia, 27100 Pavia, Italy

**Keywords:** lactic acidosis, hyperlactatemia, medications, drugs, illness, disease, d-lactic acid metabolite, l-lactic acidosis, l-lactic acidosis and disease, l-lactic acidosis and medications, acid-base equilibrium, acid-base and liver pathology

## Abstract

Lactic acidosis represents one of the most common conditions that can compromise the health of intensive care unit (ICU) patients, increasing the mortality of patients with high levels of Lactate who do not receive a proper treatment within the first 6 h of hospitalization. There are two enantiomers of lactic acid: L-lactic acid (when the concentration increases, it can lead to a state of severe acidemia risking cardiovascular collapse, causing an increase in mortality in ICU patients) and D lactic acid (produced in the human organism by microbiota and its production increases during some pathological status). Generally, increased levels of serum lactic acid could be due to numerous factors, including hypoxia (caused for example by septic/cardiogenic/hypovolemic or obstructive shock), specific pathologies (e.g., liver disease), use of some drugs (e.g., metformin), presence of toxins, and trauma. Since the underlying cause could be fatal for the ICU patient, it is important to understand the root of this clinical status with a view to correct it and prevent the risk of a poor clinical outcome. Prevention and early treatment are the keys to control the negative clinical consequences. The aim of this review is to revise the scientific literature for further confirmation about the importance of early identification of acidotic statuses and to underline how an early diagnosis can prevent the worst clinical outcome, especially for ICU patients who are more fragile compared to the general population.

## 1. Introduction

Lactic acidosis is one of the responsible factors that lead to a poor clinical outcome in ICU patients, causing a high mortality rate, while a good control of the serum lactate levels results in a decrease in morbidity and mortality. There are two enantiomers of lactic acid: L-lactic acid and D-lactic acid. The first one is associated with a large increase in mortality: it is directly correlated with the blood lactate level and it is an indicator of unbalanced glucose metabolism. If the concentration of L-lactic acid becomes too high leading to a state of severe acidemia, the main risk is cardiovascular collapse. As shown in the work of Gharipour et al., the mortality rate of ICU patients who suffered from severe hyperlactatemia (lactate level > 10 mmol/L) for more than 24 h was 96.8% [1].

D-lactic acid is produced in the human organism by microbiota and while its synthesis is controlled under normal circumstances, it increases in clinical conditions, such as short bowel syndrome and jejunoileal bypass surgery. Since infectious and inflammatory-related diseases can also cause or be associated with increased D-Lactate level, D-Lactate can be used as a marker of some infections and sepsis.

Lactic acidosis is the result of the accumulation of lactate and protons in the body fluids, and the classification of hyperlactatemia vs lactic acidosis should be conducted using arterial blood gas values, electrolyte panel and considering the history of the present illness. There are different mechanisms that can cause hyperlactatemia (consequence of the disease or due to pharmacotherapy) resulting in a classification of hyperlactatemia as type A, B, or D [2]. Type A hyperlactatemia is caused by a low tissue concentration of oxygen (hypoxia) and it can be due to several factors, including septic shock (in this case the elevated concentration of lactate is at the same time due to an increased production and a failure of clearance [3]), cardiogenic shock, hypovolemic shock, and obstructive shock. Type B hyperlactatemia is subdivided into B1 (consequence of specific diseases), B2 (related to toxins or drugs, for example biguanides and nucleoside/nucleotide reverse transcriptase inhibitors), and B3 (due to error of metabolism). Type D is the result of excessive production of D-Lactate by certain bacterial species in the gastrointestinal tract [4] and it is often seen in patients with short bowel syndrome. Delaying appropriate treatment for type A can be fatal. In fact, when lactic acidosis is due to a low-flow state or sepsis [5], it increases mortality by a factor of nearly three. Therefore, type B and D should be considered as differential diagnosis.

The most effective treatment of hyperlactatemia is the control of the underlying pathological cause. A better understanding of the different types of hyperlactatemia and a high focus on the way to treat it can help lower the mortality of ICU patients, who are the most exposed to the fatal outcome.

In this review, we wanted to survey the actual literature for further confirmation about the importance of the early identification of acidotic statuses and to compare in one paper the known facts about lactic acidosis and its consequences, especially in ICU patients.

## 2. Material and Methods

We followed the recommendation of the PRISMA-P (Preferred Reporting Items for Systematic review and Meta-analysis protocols), encoding the review on PROPSERO with serial number 363623.

A systematic review of the literature was performed, collecting the articles present in PubMed and Scopus with the following keywords: lactic acidosis, hyperlactatemia, medications, drugs, illness, disease, d-lactic acid metabolite, l-lactic acidosis, l-lactic acidosis and disease, l-lactic acidosis and medications, acid-base equilibrium, acid-base and liver pathology filtered for humans, adults (older than 18 years), language (English), and time of publication (1 January 1991 to 28 February 2021).

The studies were independently screened by two authors (TR and VF) and the disagreements between the two authors were solved by a third reviewer (CZ and YL). After identification of all the articles, we then removed: all duplicates, all articles not written in English, and all records ineligible by automatic tools, editorials, commentaries, letters to the editor, opinion articles, meeting abstracts, and original articles lacking an abstract. The relevance of the screened articles was decided upon following review of the titles and abstracts.

**Figure 1:**
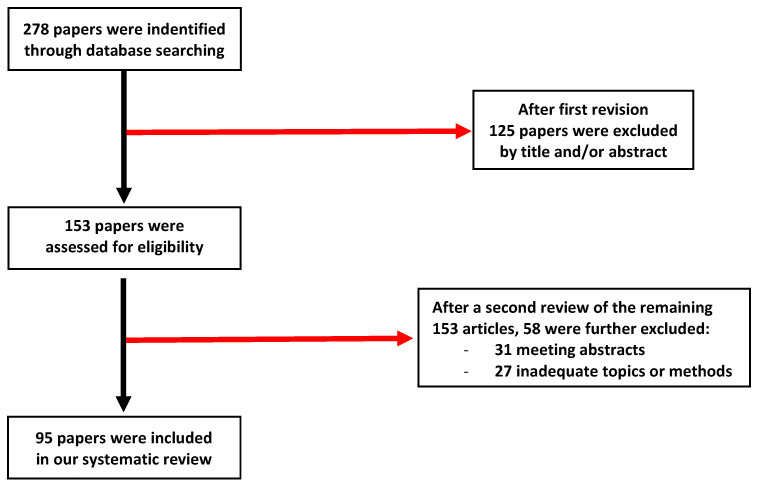
Flowchart.

Finally, we identified 153 articles, but on further analysis we selected 95 papers most belonging to the goal of our research (Figure 1).

### 2.1. Levo-Lactate and Destro-Lactate

Lactic acid is chiral and is composed of two enantiomers: L-lactic acid and D-lactic Acid.

**Figure 2:**
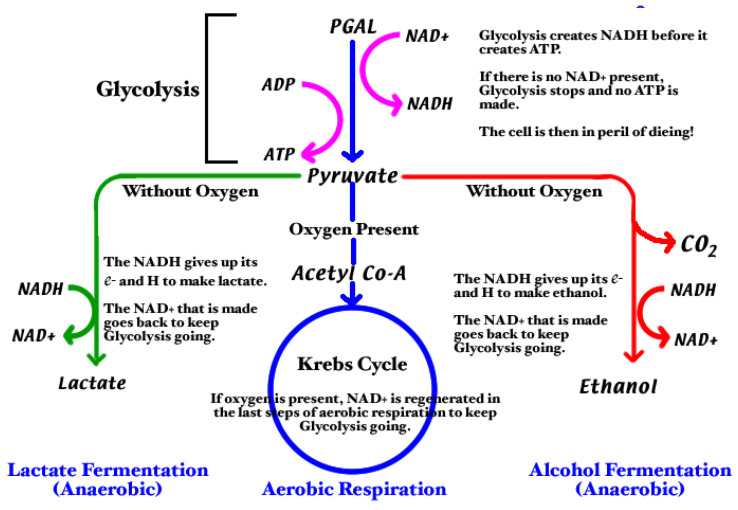
Lactate biochemical pathway. Legend: PGAL: 3-phosphoglyceraldehyde; ADP: Adenosine diphosphate; ATP: Adenosine triphosphate; NAD^+^: Nicotinamide adenine dinucleotide (oxidized form); NADH: Nicotinamide adenine dinucleotide (reduced form); CO_2_: carbon dioxide.

L-lactic acid is the organic acid that is produced from pyruvate in the process of fermentation during normal metabolism and the concentration in human blood is in a range between 0.5 and 1 mmol/L at physiological level. L-Lactate in most organisms is metabolized by L-Lactate Dehydrogenase which with NAD+ as a cofactor generates pyruvate and NADH (Figure 2).

Even though the definition for an increased level above physiological range varies in literature, L-lactic acidosis (L-LA) occurs when the L-Lactate concentration exceeds 4 mmol/L in plasma, plasma bicarbonate concentration is <20 mmol/L and blood pH drops under 7.35 [3], while a low level of L-Lactate is entitled as hypolactatemia [6] and it is a pathological state that is rarer compared to hyperlactatemia and lactic acidosis.

There are different pathological statuses that bring about hyperlactatemia, e.g., sepsis, hemorrhagic shock, cardiac arrest, trauma, poisonings, ischemia, burns, diabetic ketoacidosis, some types of cancer, and intense muscle activity [7,8,9].

The main metabolic difference between L-lactic acid and D-lactic acid is that D-lactic acid has no metabolic role in the majority of life forms [3]. The oxidation of L-Lactate is in its principle the same as the oxidation of D-Lactate by D-Lactate Dehydrogenase.

D-Lactate is produced in the human organism by microbiota, its production is controlled, and it is not a noxious compound itself. However, it can be overproduced in pathological situations, such as short bowel syndrome, jejunoileal bypass surgery, when a patient takes a meal with high sugar content [10], in a situation of abdominal compartment syndrome, or as a consequence of poisoning from heavy metals (despite the unknown mechanism that causes it) [11]. Since infectious diseases and diseases with following inflammation processes can also cause or be in relation with increased D-Lactate level [12,13,14], D-Lactate can be used as a marker of some infections and sepsis [15].

The other causes of an increase in D-Lactate level are heavy metal poisoning [16] and ingestion of contaminated food or beverages [17].

The excess of D-lactic acid, however, is not easily identified because D-Lactate and L-Lactate have the same physical and chemical properties [18], and the normal analytical methods are not able to distinguish the two isomers.

### 2.2. Levo-Lactic Acidosis

The most common cause of metabolic acidosis, especially considering the critical care setting, is L-lactic acidosis. L-lactic acidosis is associated with a large increase in mortality [1,19,20,21] and the increase in mortality is correlated directly with the blood lactate level. It is not a diagnosis but an indicator of unbalanced glucose metabolism that may be the consequence of other disorders, thus the increase in mortality is the reflection of cellular dysfunction due to deranged energy metabolism [22]. If the concentration of L-lactic acid becomes too high, leading to a state of severe acidemia, the main risk is cardiovascular collapse [23,24] and the main cause of this impairment in cellular functions may be the binding of hydrogen ions (H+) to cellular proteins.

Physiologically, L-Lactate is an intermediary in the metabolism of carbohydrate and non-essential amino acids, with a daily production of an average of 20 mmol/kg body weight [25,26]. The regeneration of adenosine triphosphate (ATP) in red blood cells takes place through glycolysis. Also producing L-lactic acid are adipose tissues [27], central nervous system, skin, muscle, and gastrointestinal tract [28]. The L-lactic acid is removed principally via gluconeogenesis in the liver (approximately 60%), in the kidney cortex (more or less 30%: the renal cut-off for the elimination of lactate is 6–10 mmol/L, thus renal excretion is significant when there is severe hyperlactatemia [29]), and for a small percentage via oxidation in many organs (liver, kidney, muscle, heart, and brain) [30]. Therefore, the accumulations of L-lactic acid can be caused by an increased rate of its production and/or a decreased rate of its removal. 

In L-LA: pathophysiology, classification, and causes; emphasis on biochemical and metabolic basis, Kamel et al. propose a classification based on the pathophysiology of L-LA, differentiating when the consequence of the disorder is the increased production and when it is a decreased removal of L-LA [31].

### 2.3. Type A: Levo-Lactic Acidosis Related to Low DO_2_

The increase in production of L-lactic acid happens when there is a mismatch between the rate of regeneration of ATP in mitochondria and the necessity of ATP to complete the biological functions. In normal metabolism, from a molecule of glucose, 2 molecules of ATP are generated during glycolysis. When there is necessity of a faster rate of ATP production, the cost is 1 mmol of L-lactic acid per 1 mmol of ATP regenerated. At first, the drop in cellular pH is controlled. However, the shortage of energy production can lead to a deficiency of function in some of the vital organs that are more sensitive to energy shortage. The therapy in patients with L-lactic acid depends on the primary cause, however the administration of sodium bicarbonate (NaHCO_3_) can be a temporary measure to direct the cause of this state [31]. Cons of this choice are that the HCO_3_^−^ reacts with H^+^ generating carbonic acid which is then converted to water and CO_2_ by carbonic anhydrase. CO_2_ enters cells more easily than bicarbonate ions, so it reduces the intracellular pH [32]. Another negative aspect is that there is an increase in blood L-Lactate level, even though this is seen as a response to the treatment since there is the H+ titrated by the bicarbonate buffer system guaranteeing the de-inhibition of PFK-1 and a rise in glycolysis activity [33]. The solution to this pathological situation is both an adequate alveolar ventilation and an adequate effective circulating volume. A continuous dialysis modality [34,35] such as veno-venous may provide the way to administrate an adequate infusion of NaHCO_3_ and contemporarily avoid the risk of fluid overload, causing pulmonary edema [36].

The two main clinical settings with overproduction of L-lactic acid are: inadequate delivery of oxygen and sepsis.

The equation that represents the global oxygen delivery (DO_2_) is:

DO_2_ = Q × Hb × 1.39 × % saturation

where Q is the cardiac output in L/min, Hb is hemoglobin concentration in g/L, and 1.39 is the Huffner’s constant representing the amount of O_2_ in mL carried per gram of hemoglobin at sea-level, when it is exposed to a PO_2_ > 97.5 mmHg.

When there is a fall in DO_2_ or there is an increase in metabolic demand, the extraction ratio of O_2_ rises to a maximum ratio of 60–70%. If there is a higher demand, the price that is paid is tissue hypoxia. The limit for tissue hypoxia varies among species, but a critical value of DO_2_ is 7.3 mL/kg/min in a human subject 19–25 years old [37] while a value of 5 mL/kg/min has been observed in an anesthetized, mechanically ventilated man with neuromuscular blockade [38]. There is variability between different organs and clinical studies have also confirmed the relationship between the development of hyperlactatemia and a supply-dependent state of O_2_ consumption [39,40].

The most common clinical events for overproduction of L-lactic acid due to reduced DO_2_ are cardiogenic shock [41], hypovolemic shock [42], severe hypoxemia (PO_2_ < 30–40 mmHg), severe euvolemic anemia (Hb < 45 g/L), and carbon monoxide poisoning (since the compound of hemoglobin and carbon monoxide is stronger than the one with oxygen).

In septic shock and sepsis with hyperlactatemia, the mechanisms behind the increase in lactate level are various. Friedman and colleagues hypothesize that there is an O_2_ debt that has to be eliminated at first by the increase in O_2_ supply, providing in this way an “early goal directed therapy” [43]. However, no other studies have been carried out to confirm such hypothesis. Another origin of the disorder can be connected to microcirculatory disfunction, or to a regional disorder of blood flow distribution, or also a defective mitochondrial oxygenation utilization [44]. It needs to be said that in critically ill patients, there is a loss in normal autoregulation of blood flow, with an increase of the activation of the coagulation cascade and an increase in red blood cell aggregation [45]. Reactive oxygen species disrupt microcirculatory function and promote platelet–endothelial adhesion [46]. The alteration of capillary permeability increases tissue edema, which further delays O_2_ extraction [47]. All these mechanisms may impair O_2_ delivery to the kidneys and liver, limiting the rate of L-lactic acid removal via gluconeogenesis.

Aside from the reduced extraction of oxygen, the cytokine cascade increases glucose uptake by cells [48], endotoxins may inactivate Pyruvate Dehydrogenase (PDH), and the activation of b2-adrenoreceptor [49] increases the production of cAMP, stimulating glycogenolysis and increasing the concentration of pyruvate, so another management of patients with L-lactic acid due to sepsis could be the b2-adrenoreceptor blockade [50], even though it would be necessary to identify patients who actually have an overactivation of b2-adrenoreceptors.

### 2.4. Type B1: Levo-Lactic Acidosis Related to Underlying Disease and Type B3: Levo-Lactic Acidosis Related to Altered Lactate Metabolism

There are clinical settings characterized with an increased production of L-lactic acid in the absence of hypoxia or an increased demand for ATP and due to the inhibition of the Electron Transport Chain: type B1 L-lactic acidosis is due to specific diseases while type B3 is a consequence of error in the metabolism of L-lactic acid.

Ethanol intoxication. The enzymes alcohol dehydrogenase and aldehyde dehydrogenase role the metabolism of ethanol, transforming NAD into NADH+H and causing a higher ratio of NADH + H/NAD leading to L-lactic acid [51]. The level of plasma lactate in patients with ethanol intoxication is usually below 5 mmol/L and is generally compensated by the ability of other organs to remove the L-lactic acid made by the liver [52]. When the severity of the acidosis increases, there is the need to look for other causes, e.g., hypoxia (due to sepsis, bleeding, pancreatitis), thiamine deficiency, and seizures (because of alcohol privation or delirium tremens) [53]. Otherwise, an underuse of L-lactic acid by the liver due to a chronic liver disease should be investigated.

Thiamine deficiency. Usually, the deficiency of thiamine relates to alcohol abuse. The union of these two factors rapidly develops L-lactic acidosis [54]. The increased production is combined with the diminished removal by other organs because of the diminished activity of PDH [55,56].

Malignancy. Patients with hematological and solid organs malignancies can develop acidosis because of the highly mitotic cells and their fast turn-over. This comes with an elevated glycolytic activity of tumor cells, tumor tissue hypoxia, and thiamine or riboflavin deficiency [57,58].

Malignancy-induced lactic acidosis (MILA), a rare paraneoplastic phenomenon, is mostly described with hematologic malignancies (lymphomas and leukemias) but has also been reported with solid tumors. It is a subset of type B lactic acidosis being mediated without evidence of tissue hypoperfusion. Lymphoma-induced lactic acidosis is often considered an oncologic emergency and is associated with an increased risk of mortality and poor prognosis. It has a complex pathophysiology centered in the “Warburg effect,” i.e., the programming of cancer cells to depend on aerobic glycolysis for promotion of their proliferation and anabolic growth. The treatment of lymphoma-induced lactic acidosis is focused on the prompt administration of chemotherapy. The role of alkali therapy in this setting is controversial and has limited proven benefit with a potential for worsening the lactic acidosis. If alkali therapy is used in the presence of severe acidemia to optimize cardiovascular status, it should be administered judiciously [59].

Congenital Levo-Lactic Acidosis. In these pathologies, L-lactic acidosis is a result of a defect in pyruvate transport from the cytosol to the mitochondrial matrix. These are genetical diseases and the genes that are mutated are mainly connected to deficiency in the function of pyruvate dehydrogenase (PDHA1) [60] or in a mitochondrial transfer RNA gene (MT-TL1) that responsible for most cases of MELAS syndrome (mitochondrial encephalomyopathy, lactic acidosis, and stroke like episodes) [60,61,62].

Diabetic Acidosis. Diabetic acidosis is a pathological status associated with Diabetes and it happens when there is hyperglycemia (blood sugar > 250/mL), metabolic acidosis (arterial pH < 7.3 and serum bicarbonate < 18 mEq) and ketosis. It is one of the most important complications of diabetes and sometimes is the first presentation of Type 1 Diabetes Mellitus. Its management aims to restore a circulatory volume, clear of ketones, and correct the electrolyte imbalance. Alongside with fluids, insulin should be administrated at 0.1 U.I/kg/h and a frequent monitoring of pH, glucose, potassium and ketones should be done [60].

Hepatopathy. The liver is one of the organs that plays a key role in the regulation of the acid-base equilibrium, in fact it is involved in the lactic acid metabolism (lactic acid is metabolized to pyruvic acid and then converted to glucose by gluconeogenesis. In the ICU, it is common to see lactic acidosis as a result of degradation of lactic acid due to tissue hypoxia or vasoconstriction), albumin homeostasis, ketogenesis, and urea production. Patients who suffer from liver cirrhosis have ABE disorders. They can develop respiratory alkalosis (most frequently), metabolic alkalosis, metabolic acidosis, respiratory acidosis, as well as a mixed disorder [60].

Methanol intoxication. The accumulation of formaldehyde and formic acid during methanol intoxication inhibits the complex IV. As a side note, severe ethylene glycol intoxication relates to falsely high blood lactate levels because of structural similarity between L-Lactate and the ethylene glycol metabolites. This happens when assays for L-Lactate use a L-Lactate oxidase reaction, while it does not if the method to measure the blood level utilizes a reaction catalyzed by lactate dehydrogenase [60]. The treatment has its focus on the administration of ethanol or fomepizole. Ethanol and fomepizole inhibit the alcohol dehydrogenase enzyme in order to stop the metabolism. Hemodialysis is necessary in order to remove methanol and formic acid [60].

Ethylene glycol intoxication. Ethylene glycol in commonly used as antifreeze solution but also as a component of e-cigarettes. The symptoms of ethylene glycol intoxication involve respectively the central nervous system (early between 0.5 and 12 h after ingestion), cardiopulmonary dysfunction (12–24 h after ingestion), and renal dysfunction (24–72 h after ingestion). Alcohol dehydrogenase starts the metabolic pathway correlated to the toxicity: ethylene glycol is oxidized to glycolaldehyde then converted to glycolic acid, glyoxylic acid and oxalic acid [61,62,63,64,65,66]. Is the oxalic acid that causes the formation of calcium oxalate crystals, which deposit in several organs and cause all the dysfunctions. Treatments are mechanical ventilation, intravenous fluids, vasopressors, calcium supplementation when hypocalcemia is present and hemodialysis when needed. The two antidotes that have to be used are methanol and fomepizole, as in methanol intoxication cases [67].

Cyanide poisoning. The main causes of cyanide exposure are inhalation from fire and occupational exposure. From a medical point of view, level of toxicity can be reached after prolonged infusion of sodium nitroprussiate, used as treatment in neurosurgical practice [59], as a treatment for hypertensive emergencies, especially if the patient is with chronic renal failure, or is a pediatric [60]. Cyanide is a small lipid soluble molecule and its target is the mitochondrial cytochrome oxidase and more specifically the iron of the heme group, inhibiting complex IV of the electron transport chain (ETC) [61]. Cyanide poisoning is treated with different antidotal therapies that have different paths of action: chelating, inducing methemoglobin, or helping to create less toxic complexes.

### 2.5. L-Lactic Acidosis Related to Medications

Usually, cases of lactic acidosis and hyperlactatemia are consequences of pathological states, but as we saw earlier in this review, they can also be due to certain therapies. Generally, the diagnosis of medication-induced hyperlactatemia or lactic acidosis (type B2 L-lactic acidosis) is a diagnosis of exclusion (Table 1).

There are medications that are well known for causing hyperlactatemia and L-lactic acidosis, and these are the ones that have an active role in the electron transport chain:

Propane 1,2-diol. It is often used as a solvent for IV and oral drugs preparation. This could sound innocuous, but since many of the drugs that it contains are often used in ICU (such as lorazepam, diazepam, barbiturates, phenytoin, trimethoprim/sulfamethoxazole [62]), the prolonged administration can result in lactic acidosis. Generally, the isomer that accumulates is the D-lactic acid since the L-lactic acid is metabolized by an alternate pathway in the liver.

Antiretroviral drugs. The prolonged use of nucleoside analogue reverse transcriptase inhibitors can lead to mitochondrial DNA depletion, impairing the electron chain transportation. In addition, the hepatic steatosis due to the mitochondrial dysfunction may decrease the removal of L-lactic acid [63,64,65,66,67].

Linezolid. The binding of linezolid with bacterial ribosomes has shown a similar mechanism with the mitochondria. In fact, linezolid [65] causes the inhibition of the synthesis of the proteins of complex IV by mitochondrial ribosomes, causing an increase in L-lactic acid [66].

Propofol. The L-lactic acidosis seems to be the consequence of an impairment of the entrance of long-chain fatty acids compared to medium and short-chain fatty acids into the mitochondria, due to the inhibition of carnitine palmityl transferase 1 by the rise in malonylcarnitine and complex II of the ETC due to the rise of C5-acylcarnirine [67].

Uncouplers of oxidative phosphorylation. When H+ exits the mitochondrial matrix and re-enters through another H+ channel or another mechanism that is not connected to the conversion of adenine diphosphate (ADP) to ATP, there is an uncoupling of the reaction of ETC and phosphorylation of ADP. Drugs that take action in this way move the H^+^ from the intermembrane space (that is the space where the concentration of H^+^ is higher) and dissipate the gradient diffusing the H^+^ into the mitochondrial matrix [68]. One of the examples of drug connected to the development of L-lactic acidosis is metformin, a biguanide that is first-line oral antihyperglycemic medication. Metformin-associated L-lactic acidosis [69] is more likely to occur in patients that have other conditions that predispose to hypoperfusion, hypoxemia, and that might have acute renal failure [70]. Acute renal failure lowers the clearance of metformin increasing the blood level > 5 ug/L. Another drug that uncouples oxidative phosphorylation is salicylic acid [71], that can cause accumulation of L-lactic acid and ketoacidosis [72,73]. In these cases, L-lactic acidosis does not have the same urgency of other kind of acidosis, since there is not overproduction and there is no problem regenerating ATP. Troubles can occur when there is also severe liver injury (might be due to acute hepatitis as a consequence of viral infection or drug toxicity), prior hypoxia, or extensive replacement of normal liver cells [74].

Besides the drugs where the mechanism is known, there are many other cases of medication-induced lactate level elevation. In a systematic review of the literature, Smith et al. [75], analyzed 1918 articles and found a total of 286 patients with medication-induced lactate level elevations. From these data, they identified 59 unique medications as causing the adverse drug effects. From their analysis, the median time that was taken to have an increased level of L-LA was three days. The patients hospitalized in the ICU were the ones that had experienced more frequently this collateral effect (46.6%). The most described medication classes are antimicrobials (n = 22), central nervous system agents (n = 10), and cardiovascular agents (n = 8), while the most reported medications were epinephrine (n = 74) and albuterol (n = 72). The only cases of fatal lactic acidosis were those secondary to the inhibition of mitochondrial protein synthesis or function (type B2 hyperlactatemia).

The elevation of lactate level has been encountered especially during treatment with two adrenergic agonists: albuterol and epinephrine. Albuterol, a drug that is especially indicated for asthma treatment, was seen as a temporary alteration with no reports of permanent alteration. Epinephrine, on the other side, is a drug that is commonly used in ICU patients and the indication varies from cardiac arrest, anaphylaxis, and shock. The administration of this drug is indicated in the most different clinical situations, so finding it among the drugs that can cause L-LA it is not a surprise [76]. Moreover, both agents stimulate the B2-adrenergic receptor, which stimulates aerobic glycolysis, generating high tissue pyruvate concentration. This is converted to lactate, so the high level of lactate could be both a secondary effect of the drug or a consequence of the underlying disease.

Organ dysfunction was present in 16.4% of patients as an acute dysfunction (acute kidney injury as the most reported), while chronic dysfunction was encountered in 14.3% of patients (73.2% end-stage liver disease).

One of the most interesting findings was that most cases happened when FDA-labeled doses were given. Therefore, when a patient develops an unexpected increase in lactate level and is receiving a drug that can cause it as an adverse reaction, the link between cause and effect should be prompt. In this case, clinicians should consider the substitution of the implicated medication, whereas possible.

### 2.6. Treatment

Treating the cause of lactic acidosis must be considered as soon as possible: administration of antibiotic agents when there is sepsis, management of cardiovascular treatment if there are arrythmias, coronary intervention for acute myocardial infarction, surgery when there are trauma or toxic megacolon, dialysis when there is the need to remove toxins or drugs or suspension of certain drugs (Table 2). When there is a lack of tissue perfusion, e.g., in patients with sepsis or hypovolemia, the usage of colloid or crystalloid solutions are effective [77], but the choice of the infusion needs to be made carefully. Saline administration can cause a non-anion gap metabolic acidosis [78] and a reduction in ionized calcium levels, causing a possible depression in cardiac function [79], while balanced salt solution can cause metabolic alkalosis [80] and a solution with high concentration of chloride is linked to acute kidney injury [81]. More studies are needed to determine the most operative crystalloid [81]. Other strategies to optimize the O_2_ delivery to tissues are red cell transfusion in order to maintain a hemoglobin concentration higher than 7 g/dL [82,83], mechanical ventilation, or invasive ventilation in particular to prevent hypercapnia.

The difficulty of catecholamines administration is that, on one side [83], acidemia causes a decreased response to the delivery of the drugs [84]. On the other side, if the catecholamines are infused at a high rate, beta2 adrenoceptor can be overstimulated and worsen hyperlactatemia. Drugs, such as dobutamine, nitroglycerin, and acetylcholine, have been found to improve the outcome of rescuing microcirculation [85].

The indication to administrate IV sodium bicarbonate for severe acidemia (pH < 7.2) is uncertain [32,86]. The administration can cause the accumulation of carbon dioxide and consequent intracellular acidification, lowering the levels of ionized calcium and altering cardiac contractility [32]. This is seen as more frequent when high quantities of bicarbonate are administered, giving no time to the lungs to remove the carbon dioxide from tissues [32]. Other buffer systems have been developed (THAM = tris-hydroylmethyl aminomethane [87] and Carbi-carb = 1:1 mixture of sodium carbonate and sodium bicarbonate) [32]. THAM is a weak base and works as a proton acceptor decreasing as well arterial PCO_2_ contributing to the formation of HCO_3_ [86,87,88,89,90,91,92,93]. It has been shown that it has a positive inotropic effect and anti-arrhythmic properties, but on the other hand alkalosis, hypoglycemia, depression of ventilation, and toxic reactions have been reported [88]. Carbicarb acts similarly to sodium bicarbonate producing smaller quantities of CO_2_ [32]. A combination of THAM, sodium bicarbonate, phosphate, and acetate (named Tribonat) was also introduced in the treatment of acidosis. It was found to be a good buffer between 6.8 and 7.4, while above 7.4 its capacity decreased [93]. However, further studies are needed to determine their possible role in the treatment of metabolic acidosis [84].

The introduction of dialysis in the treatment can be helpful, even though the rate of removal of lactic acid is lower than the rate of production when there are sever lactic acidosis states [89]. The choice is continuous dialysis rather than intermittent dialysis because of the lower rate of administration of bicarbonate and a higher tolerability in patients with hemodynamic instability [86].

As indicated by Kraut and Madias (2012), one of the factors responsible for the overload of calcium and sodium during states of lactic acidosis is the Sodium-Hydrogen exchanger NHE1, while it has been demonstrated that its inhibition reduces the damage of cells [89]. This could be the target for future therapies, as well as the inhibition of lactate dehydrogenase (LDH) and monocarboxylate (MCT) lactate transporters (responsible for the Warburg effect in cancer cells) could be the target therapy for tumor-induced systemic lactic acidosis [59,90].

The monitoring of the patient during the treatment of lactic acidosis has the aim to detect tissue hypoxia in order to assess the effectiveness of resuscitation. Assessments of hemodynamic values, oxygenation, and acid-base status are needed. The measurement of blood lactate level is the foundation for the monitoring, and it can be performed using arterial as well as venous blood [91] with an interval of 2–6 h [91]. A steadily high hyperlactatemia is linked to an increased mortality [93], while a reduction of 20% in serum lactate levels every 2 h within the first 8 h [93] of resuscitation has been associated to a decrease in morbidity and mortality.

## 3. Results and Discussion

The accumulation of lactate and protons in the body fluids leading to a state of L-lactic acidosis can result in a poor clinical outcome, increasing the mortality in ICU patients, while a reduction of 20% in serum lactate levels every 2 h under intensive care has been demonstrated to result in a decrease in morbidity and mortality. In this review, we saw that an early diagnosis can help to improve the chances of survival, allowing an early treatment (targeted to the primary cause, but also, when needed, focused on the resuscitation).

There are different types of L-lactic acidosis. One of the main challenges in early diagnosis is the distinction between D-lactic acidosis and L-lactic acidosis, but also the distinction between true hyperlactatemia or cross reaction of the reagents with the metabolites.

Type A Acidosis is due to a low concentration in tissue oxygen, and it can be a consequence of septic shock, cardiogenic shock, hypovolemic shock, obstructive shock, or trauma. Its treatment has the focus on solving the underlying cause and restoring oxygen tissue perfusion, supporting the circulation with a careful choice of the IV infusion.

Type B hyperlactatemia is subdivided into type B1 (consequence of specific diseases), type B2 (related to toxins or drugs), and type B3 (due to error of metabolism). In these cases, the main treatment is support therapy (administration of buffer system, dialysis, mechanical ventilation when needed), in order to maintain the body function and support the lowering of lactic acid.

There are future possibilities in targeted therapies, such as the inhibition of the NHE1 exchanger to reduce the damage of cells due to the overload of calcium and sodium during states of lactic acidosis as well as MCT lactate transporters in L-lactic acidosis that are tumor-induced. However, more research needs to be conducted in order to lower the impact of this pathological status.

The best outcome will depend on a tempestive diagnosis and treatment, remembering that “each lactate has its own marker and diagnostic investigation which are the first real step of the right therapy” [1,2,3,4,5,6,7,8,9,10,11,12,13,14,15,16,17,18,19,20,21,22,23,24,25,26,27,28,29,30,31,32,33,34,35,36,37,38,39,40,41,42,43,44,45,46,47,48,49,50,51,52,53,54,55,56,57,58,59,60,61,62,63,64,65,66,67,68,69,70,71,72,73,74,75,76,77,78,79,80,81,82,83,84,85,86,87,88,89,90,91,92,93].

## 4. Conclusions

Lactic acid is produced in physiologically normal processes, and as a common finding in disease states. When increased production is comorbid with decreased clearance, the severity of the clinical course escalates. Importantly, the effects of severely elevated levels of lactic acid can have profound hemodynamic consequences and can lead to death. Serum lactate levels can be both a marker for risk as well as a therapeutic target. The higher the level and the longer the time to normalization of elevated serum lactate, the greater the risk of death.

Clinicians should be aware that hyperlactatemia can occur in the presence of adequate tissue perfusion and oxygenation. Lactic acidosis on the other hand usually occurs in the presence of inadequate tissue perfusion, abnormalities in carbohydrate metabolism and with the use of certain medications.

## Figures and Tables

**Table 1:** Drugs Induced Lactic Acidosis.

Lactate Classification	Mechanism	Medication	Treatment
**Tybe A**	Reduced Cardiac Output	Clozapine, Diltiazem, Nifedipine, Procainamide	Medication Withheld Supportive Treatment Iniatiated
**Type B-1**	Drug Induced Disease	Hepatic Failure (Acetaminophen, Erlotinib, Fludarabine) Mesenteric Ischemia (Canaglifozin) Gastrointestinal Ischemia (Epinephrine) Pancreatitis (Cimetidine, Exetanide, Rifampin, Isoniazid, Pyrazinamide, Ethambutol) Acute Kidney Injury (Exetanide, Ibuprofen, NSAID) Hemolytic Anemia (Sulfamethoxazole and Trimethoprim, Nitrofurantoin) Rhabdomyolysis (ralgtegravir) Hypoxiemia (Opioids)	Medication Withheld Antidotes Administered If Applicable Supportive Treatment Initiated
**Type B-2** **Related to** **Metabolism** **Impairment**	Increased Pyruvate Production	Albuterol, Metaproterenol, Salmeterol, Sorbitol, Caffeine, Theophylline, Epinephrine, Terbutaline	Medication Withheld AntidotesAdministered If Applicable Supportive Treatment Initiated
	Alterations in Glucose Metabolism Favoring Accumulation of Lactate Relative to Pyruvate	Ethanol, Niacin, Oxaliplatin, Streptozocin	Medication Withheld Supportive Treatment Initiated
	Propylene Glycol excipient	Diazepam, Lorazepam, Etomidate, Pentobarbital, Sulfamethoxazole and Trimethoprim, Nitroglycerin	Medication Withheld Supportive Treatment Initiated
**Type B-2** **related to** **mitochondrial impairment**	Inhibition of Mitochondrial Protein Synthesis/Function	Acetaminophen, Carboplatin, Linezolid, Simvastatin, Minocycline, Telbivudine, Adefovir, Entecavir, Linezoild	Medication Withheld Supportive Treatment Initiated
	Inhibition of Mitochondrial Electron Transport Chain	Clozapine, Propofol, Sodium Nitroprusside, Valproate, Venlafaxine	Medication Withheld Supportive Treatment Initiated
	Mitochondrial Toxicity	Ganciclovir, Valanciclovir, Ribavirin-Sofosbuvir, Adasabuvir.Ombitasvir, Paritaprevir, Ritonavir	Medication Withheld Supportive Treatment Initiated
	Uncoupling Oxidative Phosphorylation	Sulfasalazine, Metformin	Medication Withheld Supportive Treatment Initiated
**Type D**	Bacterial Overgrowth in Setting of Short Bowel Syndrome	Doxycicline, Sulfamethoxazole and Trimethoprim	Medication Withheld Supportive Treatment Initiated

**Table 2:** Types of Acidosis.

Type of Acidosis	Mechanism	Causes	Therapies
**Type A**	Mismatch VO_2_(O_2_ Consumption) DO_2_(O_2_ Delivery)	Any Shock(Septic, Cardiogenic, Hypovolemic, Obstructive) Trauma Severe Hypoxemia Carbon Monoxide Infection	Adequate Alveolar Ventilation Adequate Circulation Volume Continuous Dyalisis Antibiotics Cardiovascular Treatment Withheld Medications
**Type B-1**	Related to Specific Disease	Hepatopathy Thiamine Deficiency Malignancy Cyanide Poisoning	Stop Drug Administration Support Therapy
**Type B-2** **Type B-3**	Related to Toxins or Drugs Related to Metabolism Alterations	See Table 1 Congenital L-LA Ethanol Intoxication Methanol Intoxication	See Table 1 Support Therapy
**Type D**	d-Lactate Excessive production	Short Bowel Syndrome	Surgery and Support

## Data Availability

Not applicable.

## References

[1] Gharipour A., Razavi R., Gharipour M., Modarres R., Nezafati P., Mirkheshti N. (2021). The incidence and outcome of severe hyperlactatemia in critically ill patients. Intern. Emerg. Med..

[2] Piccioni A., Saviano A., Cicchinelli S., Valletta F., Santoro M.C., de Cunzo T., Zanza C., Longhitano Y., Tullo G., Tilli P. (2021). Proadrenomedullin in Sepsis and Septic Shock: A Role in the Emergency Department. Medicina.

[3] Gunnerson K.J., Saul M., He S., Kellum J.A. (2006). Lactate versus non-lactate metabolic acidosis: A retrospective outcome evaluation of critically ill patients. Crit. Care.

[4] Pohanka M. (2020). D-Lactic Acid as a Metabolite: Toxicology, Diagnosis, and Detection. Biomed Res. Int..

[5] Evans O.B., Stacpoole P.W. (1982). Prolonged hypolactatemia and increased total pyruvate dehydrogenase activity by dichloroacetate. Biochem. Pharmacol..

[6] Merrells R.J., Cripps A.J., Chivers PTFournier P.A. (2019). Role of lactic acidosis as a mediator of sprint-mediated nausea. Physiol. Rep..

[7] Theobald J., Schneider J., Cheema N., DesLauriers C. (2020). Time to development of metformin-associated lactic acidosis. Clin. Toxicol..

[8] Zanza C., Thangathurai J., Audo A., Muir H.A., Candelli M., Pignataro G., Thangathurai D., Cicchinelli S., Racca F., Longhitano Y. (2019). Oxidative stress in critical care and vitamins supplement therapy: “a beneficial care enhancing”. Eur. Rev. Med. Pharm. Sci..

[9] Petersen C. (2017). D-lactic acidosis. Nutr. Clin. Pract..

[10] Fabian E., Kramer L., Siebert F., Högenauer C., Raggam R.B., Wenzl H., Krejs G.J. (2017). D-Lactic acidosis-case report and review of the literature. Z. Für. Gastroenterol..

[11] Alarcon P., Hidalgo A.I., Manosalva C., Cristi R., Teuber S., Hidalgo M.A., Burgos R.A. (2019). Metabolic disturbances in synovial fluid are involved in the onset of synovitis in heifers with acute ruminal acidosis. Sci. Rep..

[12] Naik P., Singh S., Dave V.P., Ali M.H., Kumar A., Joseph J. (2020). Vitreous d-lactate levels as a biomarker in the diagnosis of presumed infectious culture negative endophthalmitis. Curr. Eye Res..

[13] Terpstra M.L., Sinnige M., Hugenholtz F., Peters-Sengers H., Remmerswaal E.B.M., Geerlings S.E., Bemelman F.J. (2019). Butyrate production in patients with end-stage renal disease. Int. J. Nephrol. Renov. Dis..

[14] Jorgensen V.L., Reiter N., Perner A. (2006). Luminal concentrations of L- and D-lactate in the rectum may relate to severity of disease and outcome in septic patients. Crit. Care.

[15] Huang Y.S., Li Y.C., Tsai P.Y., Lin C.-E., Chen C.-M., Chen S.-M., Lee J. (2017). Accumulation of methylglyoxal andd-lactate in Pb-induced nephrotoxicity in rats. Biomed. Chromatogr..

[16] Geissler A.J., Behr J., von Kamp K., Vogel R.F. (2016). Metabolic strategies of beer spoilage lactic acid bacteria in beer. Int. J. Food Microbiol..

[17] Andersen L.W., Mackenhauer J., Roberts J.C., Berg K.M., Cocchi M.N., Donnino M.W. (2013). Etiology and therapeutic approach to elevated lactate levels. Mayo Clin. Proc..

[18] Liu Z., Meng Z., Li Y., Zhao J., Wu S., Gou S., Wu H. (2019). Prognostic accuracy of the serum lactate level, the SOFA score and the qSOFA score for mortality among adults with Sepsis. Scand. J. Trauma Resusc. Emerg. Med..

[19] Van Beest P.A., Brander L., Jansen S.P., Rommes J.H., Kuiper M.A., Spronk P.E. (2013). Cumulative lactate and hospital mortality in ICU patients. Ann. Intensive Care.

[20] Pro C.I., Yealy D.M., Kellum J.A., Huang D.T., Barnato A.E., Weissfeld L.A., Pike F., Terndrup T., Wang H.E., Hou P.C. (2014). A randomized trial of protocol-based care for early septic shock. N. Engl. J. Med..

[21] Kliegel A., Losert H., Sterz F., Holzer M., Zeiner A., Havel C., Laggner A.N. (2004). Serial lactate determinations for prediction of outcome after cardiac arrest. Medicine.

[22] Mitchell J.H., Wildenthal K., Johnson R.L. (1972). The effects of acid-base disturbances on cardiovascular and pulmonary function. Kidney Int..

[23] Davies A.O. (1984). Rapid desensitization and uncoupling of human betaadrenergic receptors in an in vitro model of lactic acidosis. J Clin. Endocrinol. Metab..

[24] van Hall G. (2010). Lactate kinetics in human tissues at rest and during exercise. Acta Physiol..

[25] Connor H., Woods H.F. (1982). Quantitative aspects of L(þ)-lactate metabolism in human beings. Ciba Found. Symp..

[26] Jansson P.A., Smith U., Lonnroth P. (1990). Evidence for lactate production by human adipose tissue in vivo. Diabetologia.

[27] Wilson T.H. (1956). The role of lactic acid production in glucose absorption from the intestine. J. Biol. Chem..

[28] Bellomo R. (2002). Bench-to-bedside review: Lactate and the kidney. Crit. Care.

[29] Connor H., Woods H.F., Ledingham J.G., Murray J.D. (1982). A model of L(þ)- lactate metabolism in normal man. Ann. Nutr. Metab..

[30] Kamel SKamel Man SOh Mitchell L. (2020). Halperin, L-lactic acidosis: Pathophysiology, classification, and causes; emphasis on biochemical and metabolic basis. Kidney Int..

[31] Ritter J.M., Doktor H.S., Benjamin N. (1990). Paradoxical effect of bicarbonate on cytoplasmic pH. Lancet.

[32] Halperin M.L., Cheema-Dhadli S., Halperin F.A., Kamel K.S. (1994). Rationale for the use of sodium bicarbonate in a patient with lactic acidosis due to a poor cardiac output. Nephron.

[33] Cerda J., Tolwani A.J., Warnock D.G. (2012). Critical care nephrology:management of acid-base disorders with CRRT. Kidney Int..

[34] De Corte W., Vuylsteke S., De Waele J.J., Dhondt A.W., Decruyenaere J., Vanholder R., Hoste E.A.J. (2014). Severe lactic acidosis incritically ill patients with acute kidney injury treated with renal replacement therapy. J. Crit. Care.

[35] Gowrishankar M., Kamel K.S., Halperin M.L. (2007). A brain protein centered view of Hþ buffering. J. Am. Soc. Nephrol..

[36] Lieberman J.A., Weiskopf R.B., Kelley S.D., Feiner J., Noorani M., Leung J., Toy P., Viele M. (2000). Critical oxygen delivery in conscious humans is less than 7.3 ml O_2_ × kg^−1^ × min^−1^. Anesthesiology.

[37] Van Woerkens E.C., Trouwborst A., van Lanschot J.J. (1992). Profound hemodilution: What is the critical level of hemodilution at which oxygen delivery-dependent oxygen consumption starts in an anesthetized human?. Anesth. Analg..

[38] Zhang H., Vincent J.L. (1993). Oxygen extraction is altered by endotoxin during tamponade-induced stagnant hypoxia in the dog. Circ. Shock..

[39] Nathan A.T., Singer M. (1999). The oxygen trail: Tissue oxygenation. Br. Med. Bull..

[40] Chiolero R.L., Revelly J.P., Leverve X., Gersbach P., Cayeux M., Berger M.M., Tappy L. (2000). Effects of cardiogenic shock on lactate and glucose metabolism after heart surgery. Crit. Care Med..

[41] Akkose S., Ozgurer A., Bulut M., Koksal O., Ozdemír F., Ozguç H. (2007). Relationships between markers of inflammation, severity of injury, and clinical outcomes in hemorrhagic shock. Adv. Ther..

[42] Friedman G., De Backer D., Shahla M., Vincent J.L. (1998). Oxygen supply dependency can characterize septic shock. Intensive Care Med..

[43] Singel D.J., Stamler J.S. (2005). Chemical physiology of blood flow regulation by red blood cells: The role of nitric oxide and S-nitrosohemoglobin. Annu. Rev. Physiol..

[44] Revelly J.P., Ayuse T., Brienza N., Fessler H.E., Robotham J.L. (1996). Endotoxic shock alters distribution of blood flow within the intestinal wall. Crit. Care Med..

[45] Cerwinka W.H., Cooper D., Krieglstein C.F., Ross C.R., McCord J.M., Granger D.N. (2003). Superoxide mediates endotoxin-induced platelet-endothelial cell adhesion in intestinal venules. Am. J. Physiol. Heart Circ. Physiol..

[46] Fink M.P. (2003). Intestinal epithelial hyperpermeability: Update on the pathogenesis of gut mucosal barrier dysfunction in critical illness. Curr Opin Crit. Care.

[47] Taylor D.J., Faragher E.B., Evanson J.M. (1992). Inflammatory cytokines stimulate glucose uptake and glycolysis but reduce glucose oxidation in human dermal fibroblasts in vitro. Circ. Shock..

[48] Kraut J.A., Madias N.E. (2016). Lactic acidosis: Current treatments and future directions. Am. J. Kidney Dis..

[49] Fulop M., Bock J., Ben-Ezra J., Antony M., Danzig J., Gage J.S. (1986). Plasma lactate and 3-hydroxybutyrate levels in patients with acute ethanol intoxication. Am. J. Med..

[50] MacDonald L., Kruse J.A., Levy D.B., Marulendra S., Sweeny P.J. (1994). Lactic acidosis and acute ethanol intoxication. Am. J. Emerg. Med..

[51] Donnino M.W., Vega J., Miller J., Walsh M. (2007). Myths and misconceptions of Wernicke’s encephalopathy: What every emergency physician should know. Ann. Emerg. Med..

[52] Shull P.D., Rapoport J. (2010). Life-threatening reversible acidosis caused by alcohol abuse. Nat. Rev. Nephrol..

[53] Klein M., Weksler N., Gurman G.M. (2004). Fatal metabolic acidosis caused by thiamine deficiency. J. Emerg. Med..

[54] Neale B.W., Mesler E.L., Young M., Rebuck J.A., Weise W.J. (2005). Propylene glycol-induced lactic acidosis in a patient with normal renal function: A proposed mechanism and monitoring recommendations. Ann. Pharm..

[55] Kelner M.J., Bailey D.N. (1985). Propylene glycol as a cause of lactic acidosis. J. Anal. Toxicol..

[56] Arbour R., Esparis B. (2000). Osmolar gap metabolic acidosis in a 60-year-old man treated for hypoxemic respiratory failure. Chest.

[57] Duriseti P., Moreno Vanegas Y., Jaber B.L., Balakrishnan V.S., Madias N.E. (2021). Malignancy-induced lactic acidosis in adult lymphoma. Clin. Nephrol..

[58] Yang R., Wang Z., Jia Y., Li H., Mou Y. (2022). Comparison of Clinical Efficacy of Sodium Nitroprusside and Urapidil in the Treatment of Acute Hypertensive Cerebral Hemorrhage. J. Healthc. Eng..

[59] Tinker J.H., Michenfelder J.D. (1976). Sodium nitroprusside: Pharmacology, toxicology and therapeutics. Anesthesiology.

[60] Borron S.W., Baud F.J. (2012). Antidotes for acute cyanide poisoning. Curr. Pharm. Biotechnol..

[61] Bulathsinghala M., Keefer K., Van de Louw A. (2016). Trimethoprim/sulfamethoxazole-induced severe lactic acidosis: A case report and review of the literature. Medicine.

[62] Schramm C., Wanitschke R., Galle P.R. (1999). Thiamine for the treatment of nucleoside analogue-induced severe lactic acidosis. Eur. J. Anaesthesiol..

[63] Luzzati R., Del Bravo P., Di Perri G., Luzzani A., Concia E. (1999). Riboflavine and severe lactic acidosis. Lancet.

[64] Garrabou G., Soriano A., Lopez S., Guallar J.P., Giralt M., Villarroya F., Martiínez J.A., Casademont J., Cardellach F., Mensa J. (2007). Reversible inhibition of mitochondrial protein synthesis during linezolid-related. Antimicrob. Agents Chemother..

[65] Soriano A., Miro O., Mensa J. (2005). Mitochondrial toxicity associated with linezolid. N. Engl. J. Med..

[66] Wolf A., Weir P., Segar P., Stone J., Shield J. (2001). Impaired fatty acid oxidation in propofol infusion syndrome. Lancet.

[67] Inzucchi S.E., Bergenstal R.M., Buse J.B., Diamant M., Ferrannini E., Nauck M., Peters A.L., Tsapas A., Wender R., Matthews D.R. (2012). Management of hyperglycemia in type 2 diabetes: A patient-centered approach: Position statement of the American Diabetes Association (ADA) and the European Association for the Study of Diabetes (EASD). Diabetes Care.

[68] Misbin R.I. (2004). The phantom of lactic acidosis due to metformin in patients with diabetes. Diabetes Care.

[69] Laforest C., Saint-Marcoux F., Amiel J.B., Pichon N., Merle L. (2013). Monitoring of metformininduced lactic acidosis in a diabetic patient with acute kidney failure and effect of hemodialysis. Int. J. Clin. Pharm. Ther..

[70] Gabow P.A., Anderson R.J., Potts D.E., Schrier R.W. (1978). Acid-base disturbances in the salicylate-intoxicated adult. Arch. Intern. Med..

[71] Jorgensen T.G., Weis-Fogh U.S., Nielsen H.H., Olesen H.P. (1976). Salicylate- and aspirin-induced uncoupling of oxidative phosphorylation in mitochondria isolated from the mucosal membrane of the stomach. Scand. J. Clin. Lab. Investig..

[72] Silvestre J., Carvalho S., Mendes V., Coelho L., Tapadinhas C., Ferreira P., Povoa P., Ceia F. (2007). Metformin-induced lactic acidosis: A case series. J. Med. Case Rep..

[73] Heinig R.E., Clarke E.F., Waterhouse C. (1979). Lactic acidosis and liver disease. Arch. Intern. Med..

[74] Smith Z.R., Horng M., Rech M.A. (2019). Medication-Induced Hyperlactatemia and Lactic Acidosis: A Systematic Review of the Literature. Pharmacotherapy.

[75] Link M.S., Berkow L.C., Kudenchuk P.J., Halperin H.R., Hess E.P., Moitra V.K., Neumar R.W., O’Neil B.J., Paxton J.H., Silvers S.M. (2015). Part 7: Adult advanced cardiovascular life support: 2015 American Heart Association Guidelines Update for Cardiopulmonary Resuscitation and Emergency Cardiovascular Care. Circulation.

[76] Severs D., Hoorn E.J., Rookmaaker M.B. (2014). A critical appraisal of intravenous fluids: From the physiological basis to clinical evidence. Nephrol. Dial. Transpl..

[77] O’Dell E., Tibby S.M., Durward A., Murdoch I.A. (2007). Hyperchloremia is the dominant cause of metabolic acidosis in the postresuscitation phase of pediatric meningococcal sepsis. Crit. Care Med..

[78] Cooper D.J., Walley K.R., Wiggs B.R., Russell J.A. (1990). Bicarbonate does not improve hemodynamics in critically ill patients who have lactic acidosis: A prospective, controlled clinical study. Ann. Intern. Med..

[79] Myburgh J.A., Mythen M.G. (2013). Resuscitation fluids. N. Engl. J. Med..

[80] Yunos N.M., Bellomo R., Hegarty C., Story D., Ho L., Bailey M. (2012). Association between a chloride-liberal vs chloriderestrictive intravenous fluid administration strategy and kidney injury in critically ill adults. JAMA.

[81] Kraut J.A., Madias N.E. (2014). Lactic acidosis. N. Engl. J. Med..

[82] Vincent J.L., De Backer D. (2013). Circulatory shock. N. Engl. J. Med..

[83] Kraut J.A., Madias N.E. (2012). Treatment of acute metabolic acidosis: A pathophysiologic approach. Nat. Rev. Nephrol..

[84] De Backer D., Creteur J., Dubois M.J., Sakr Y., Koch M., Verdant C., Vincent J. (2006). The effects of dobutamine on microcirculatory alterations in patients with septic shock are independent of its systemic effects. Crit. Care Med..

[85] Jung B., Rimmele T., Le Goff C., Chanques G., Corne P., Jonquet O., Muller L., Lefrant J.-Y., Guervilly C., Papazian L. (2011). Severe metabolic or mixed acidemia on intensive care unit admission: Incidence, prognosis and administration of buffer therapy: A prospective, multiple-center study. Crit. Care.

[86] Bjerneroth G. (1999). Tribonat--a comprehensive summary of its properties. Crit. Care Med..

[87] Hood V.L., Tannen R.L. (1998). Protection of acid–base balance by pH regulation of acid production. N. Engl. J. Med..

[88] Okorie O.N., Dellinger P. (2011). Lactate: Biomarker and potential therapeutic target. Crit. Care Clin..

[89] Kruse O., Grunnet N., Barfod C. (2011). Blood lactate as a predictor for in-hospital mortality in patients admitted acutely to hospital: A systematic review. Scand. J. Trauma Resusc. Emerg. Med..

[90] Lee S.W., Hong Y.S., Park D.W., Choi S., Moon S., Park J., Kim J., Baek K. (2008). Lactic acidosis not hyperlactatemia as a predictor of in hospital mortality in septic emergency patients. Emerg. Med. J..

[91] Jansen T.C., van Bommel J., Schoonderbeek F.J., Visser S.J.S., van der Klooster J.M., Lima A.P., Willemsen S.P., Bakke J. (2010). Early lactate-guided therapy in intensive care unit patients: A multicenter, open-label, randomized controlled trial. Am. J. Respir. Crit. Care Med..

[92] Longhitano Y., Zanza C., Thangathurai D., Taurone S., Kozel D., Racca F., Audo A., Ravera E., Migneco A., Piccioni A. (2020). Gut Alterations in Septic Patients: A Biochemical Literature Review. Rev. Recent Clin. Trials..

[93] Piccioni A., Valletta F., Zanza C., Esperide A., Franceschi F. (2020). Novel biomarkers to assess the risk for acute coronary syndrome: Beyond troponins. Intern. Emerg. Med..

